# Prophylactic Swallow Therapy for Patients with Head and Neck Cancer Undergoing Chemoradiotherapy: A Randomized Trial

**DOI:** 10.1007/s00455-017-9790-6

**Published:** 2017-04-25

**Authors:** Barbara Pisano Messing, Elizabeth C. Ward, Cathy L. Lazarus, Melissa Kim, Xian Zhou, Jessica Silinonte, Dorothy Gold, Karen Harrer, Karen Ulmer, Samantha Merritt, Geoffrey Neuner, Marshall Levine, Ray Blanco, John Saunders, Joseph Califano

**Affiliations:** 1 The Milton J Dance, Jr. Head and Neck Center, Johns Hopkins Head & Neck Surgery, Johns Hopkins Voice Center, 6569 N. Charles Street, PPW Suite 401, Baltimore, MD 21204 USA; 2 School of Health and Rehabilitation Sciences, The University of Queensland, Brisbane, QLD Australia; 3 Centre for Functioning and Health Research, Queensland Government, Brisbane, QLD Australia; 4 Icahn School of Medicine at Mount Sinai, Thyroid Head and Neck Research Center, Thyroid Head and Neck Cancer (THANC) Foundation, Department of Otolaryngology Head & Neck Surgery, Mount Sinai Beth Israel, New York, USA; 5 The Division of Biostatistics and Bioinformatics at the Department of Oncology, School of Medicine, Johns Hopkins University, Baltimore, USA; 6 Department of Otolaryngology-Head and Neck Surgery, Johns Hopkins Medical Institutions, Baltimore, MD USA

**Keywords:** Dysphagia, Swallowing, Chemoradiation, Prophylactic exercises, Quality of life

## Abstract

Evidence supporting prophylactic swallow exercises for patients with head and neck cancer (HNC) has not been universally demonstrated. This RCT examined diet level, feeding tube use, swallow function, and quality of life (QOL) of patients undergoing chemoradiotherapy who performed prophylactic swallowing exercises. Sixty HNC patients were randomized into exercise versus control groups. Swallowing, oromotor, toxicity, and QOL data were recorded (baseline, 3, 6, 12, 24 months). Physiological swallow function was examined at baseline and 3 months. Swallow exercises were completed twice daily. Oral intake at 3 months was 10% better in the exercise group, which was not statistically significant (*p* = 0.49). Significant (*p* < 0.05) differences in secondary outcomes including oromotor function, pharyngeal impairment, oral pharyngeal swallow efficiency, and incisal opening were noted at early time points (3–6 months) in the exercise group. Possible positive early improvements in swallow function are associated with swallowing exercises, although these improvements are not significant longer term.

## Introduction

The National Institute of Dental and Craniofacial Research (NIDCR, 2015) estimates those side effects during and post-treatment will occur in almost all participants receiving radiation to the head and neck area [1]. Treatment-induced toxicities including mucositis, xerostomia, odynophagia, trismus, hypogeusia/dysgeusia, as well as the potential for infections, have a significant impact on swallow function [2–8]. Radiation therapy also alters tissue integrity, leading to fibrosis [9]. Fibrosis impacts both the oral and pharyngeal muscles, contributing to reduced mobilization of muscles and structures during swallowing. The addition of adjuvant chemotherapy as a radiosensitizer to the treatment regimen is recognized to exacerbate the severity of dysphagia. Recent studies have investigated the detrimental effects of progressive neuropathy post radiation in the head and neck cancer patient [10, 11]. Significant radiation-associated dysphagia termed as late-RAD show worsening dysphagia sometimes developing years post-treatment. Awan et al. [11] reported that progressive neuropathy resulting in significant dysphagia is associated with delayed negative effects to lower cranial nerves (IX, X, and XIII).

The primary adverse effects of chemoradiation (CRT) impacts key anatomical areas and may result in weakness of the base of tongue, prolonged pharyngeal transit time, lack of coordination between swallowing phases, reduced elevation of the larynx, and reduced laryngeal closure and epiglottic inversion. These adverse effects contribute to a high rate of aspiration [7, 12], resulting in the need for alternative methods of nutrition and hydration either temporarily or in the long term [13–16].

Despite increased awareness of the importance of early supportive care for dysphagia management, recent literature confirms that access to services and management of dysphagia during and post-treatment continues to be an issue for patients internationally [17–20]. Historically, individuals with head and neck cancer (HNC) undergoing CRT as primary treatment were referred to a speech-language pathologist (SLP) for evaluation and treatment of their presenting swallow deficits months or years post-treatment. Given the delay in referring patients to speech pathology services, rehabilitation of swallow was more difficult due to the increased severity levels of dysphagia and PEG tube dependency.

As it is generally agreed that prolonged disuse is detrimental to swallow function in the HNC population [21], there has been increasing interest in the potential benefit of prophylactic swallowing interventions conducted intensively during and early post-treatment to reduce dysphagia in patients treated with primary CRT [4, 22–30]. Several studies have found some positive results with varying degrees in functional outcomes [4, 22–26, 28, 29]. Specifically, some of these studies suggested that patients who underwent CRT and adhered to a swallowing exercise program during and/or after treatment returned to an oral diet sooner, had improved weight gain, shorter duration of gastrostomy tube use, and/or exhibited higher quality of life scores [22, 24–27, 29, 30]. Additionally, less floor of mouth muscle deterioration with the implementation of prophylactic swallow exercise protocols has been shown [22].

However, many of these studies have weak study designs (retrospective studies, small cohort studies), and two systematic literature reviews examining swallow physiology in HNC participants noted that the exercise protocols used across studies to date were highly variable [19, 27]. It also must be noted that few studies have found the same positive effects across the many data points monitored. Other recent research found no improvement in function with prophylactic swallow protocols though it was acknowledged that the randomized controlled trial (RCT) was small and underpowered [31].

Due to inconsistencies in the current literature, further research is warranted to investigate the potential benefits of providing prophylactic swallow exercises for patients with HNC undergoing organ preservation treatment. Therefore, the aim of the current study was to determine whether intensive prophylactic swallow interventions provided to patients with HNC undergoing CRT would result in better swallowing outcomes and improved quality of life as compared to a control group (no treatment). The primary hypothesis was that the exercise group would demonstrate superior functional swallowing outcomes at 3 months post-treatment. Secondary hypotheses were that the exercise group would demonstrate superior physiological swallow function, shorter gastrostomy tube dependency duration, and improved quality of life over a 24 month period.

## Methods

### Randomization

Study recruitment was performed by a clinical research associate who was independent of administration of the assessment and treatment protocol. Once consented, the clinical research associate randomized participants into an exercise or control group according to a predetermined single block, computer-generated randomization schedule originated by the study statistician. Study investigators were blinded to the randomization schedule. The speech pathologist and study participants were aware which group they were assigned. The attending head and neck surgeons, medical and radiation oncologists were blinded to the randomization assignment of their patients. All clinicians involved in the management of consented patients were aware of their participation in this trial, and the procedures as set out in the study protocol for assessment and management were followed.

### Participants

Eligible participants were recruited through a tertiary head and neck cancer center in Baltimore, Maryland, USA, beginning in December 2004, closing to recruitment in May 2011 and ending data collection in August 2013. Following Institutional Review Board approval, eligible participants were identified at a weekly head and neck tumor board conference by the speech pathologist. Participants were required to be at least 21 years of age with biopsy-proven Stage III or IV squamous cell carcinoma of the oral, oropharynx, pharynx or larynx regions only to meet inclusion criteria. Only those participants who were scheduled to receive curative non-surgical treatment at the institution, and were planned for completion of combined chemotherapy and hyperfractionated radiation therapy adhering with the institution’s organ preservation protocol for Stage III-IV HNC (using traditional 3D planning techniques with concomitant cisplatin and 5-fluorouracil) were recruited in the first few years. In the final years of recruitment, due to a change within the clinic away from this protocol, only those then planned to receive the modified organ preservation protocol for Stage III-IV HNC (using IMRT inverse planning technology and delivery used image guidance with concomitant cisplatin) were recruited. Recruitment was confined to these protocols only to minimize variability from different treatment regimes.

In addition, the physicians assessed the participants’ ability to perform ordinary tasks using the Karnofsky Performance Status Scale [32], with a score of 50% or more required for participation. Participants were excluded if there was evidence of distant disease, previous radiation therapy, major psychiatric illness, severe dysphagia before treatment requiring parenteral nutrition, or any concurrent illness that, in the investigator’s judgment, might increase the risk associated with participation in the study.

### CRT and PEG Protocol

During the study period, the center’s Organ Preservation Protocol for Stage III-IV head and neck cancer consisted of one of two curative-intent cisplatin-based chemoradiotherapy regimens (Regimen 1 and 2), due to a change in the clinics management protocols mid-way through the study. The transition from Regimen 1 to Regimen 2 occurred at the same time for both participants groups in this study and analysis confirmed no statistically significant difference in proportions of patients who received Regime 1 or 2 in either group. All patients received hyperfractionated radiation at a dose of 125 cGy delivered twice daily for 28-33 days for a total dose of 70 Gy (67-72 Gy) to the primary tumor site, 60 Gy to the entire ipsilateral and/or contralateral neck and/or supraclavicular region if they contained involved lymph nodes, and 50 Gy to an uninvolved ipsilateral neck and/or contralateral neck and supraclavicular regions. The radiation therapy used in Regimen 1 was planned using traditional 3D planning techniques, whereas Regimen 2 was planned using IMRT inverse planning technology and delivery used image guidance. All patients received a one-week treatment break after delivery of 40 Gy of radiotherapy. With chemotherapy, Regimen 1 involved concomitant cisplatin (12 mg/m2 over 1 h) and 5-fluorouracil (600 mg/m2 over 20 h), dosed daily for five consecutive days during weeks one and six of radiation therapy. Regimen 2 involved concomitant cisplatin 40 mg/m2 weekly for 6 infusions.

All participants had a PEG tube placed at least one week before treatment as per the usual standard of care in this institution. However, PEG use was not commenced until participants had difficulty maintaining adequate nutrition and hydration from an oral diet alone.

Participants’ intake and body weight were monitored weekly using their weight measured to calculate chemotherapy dosage, or more frequently if issues arose. Participants with self-reported decreased oral intake, 2–3 weeks of continued weight loss, severe odynophagia, dysgeusia and/or dysphagia initiated tube feeds in combination with ongoing oral intake based on recommendations, guidance, and instruction from the dietitian, oncology nurse, and/or speech pathologist. PEG use in this study was recorded from the time non-oral feeding commenced, not from the time of PEG insertion. All patients were encouraged to continue to eat and drink, as tolerated during CRT even after PEG use began. PEG removal occurred post-treatment when participants resumed p.o. intake with weight gain or stable weight and no PEG use over a 2–4 week period. The decision for PEG removal was made by the physician, patient and often included input from the dietitian and speech pathologist based on the above criteria.

Participants underwent a PET/CT at 8–12 weeks post-treatment. Participants with N2 or greater neck disease or positive findings on the PET/CT underwent a post-treatment neck dissection at 8–12 weeks post-treatment.

### Study Protocol

The RCT consisted of two arms including an exercise and control group. Participants in both groups were assessed across a battery of measures at baseline and 3, 6, 12, and 24 months. The assessment battery is detailed below.

#### Assessment Battery

The assessment protocol completed by all participants included clinician-reported outcomes and patient-reported outcomes at each assessment time point. The primary outcome variable was functional oral intake at 3 months, as assessed using the Functional Oral Intake Scale (FOIS) [33]. FOIS ratings were made at all study time points. The SLP recorded and classified the nature of oral intake of foods (1 = normal/regular diet, 2 = soft/easy to chew foods, 3 = blenderized/pureed, 4 = non oral/PEG tube + some blenderized/pureed foods, 5 = non oral/PEG tube dependent) and fluids (1 = normal, 2 = nectar, 3 = honey thick, 4 = pudding thick, 5 = no liquids by mouth/PEG tube dependent) that were managed safely at each time point as determined by patient report, information from the patients’ medical record and/or direct observation of food and liquid intake during a clinical swallow evaluation or swallow therapy session. This functional food/fluid level information was used to determine FOIS scores. As all patients had a PEG inserted prophylactically, the FOIS score accounted for tube feeding *only if in use* i.e. the FOIS score reflected non-oral feeding only if this had commenced. Similarly, once a patient had ceased non-oral feeding but may have had the PEG still insitu, the FOIS score reflected their oral intake status only. Presence or absence of a feeding tube at each time point was documented, as was overall duration that the gastrostomy tube remained in place.

Oromotor function was measured using an oromotor assessment containing 69 tasks assessing strength and range of motion of the facial muscles, tongue, and palate; and included measurement of incisal opening using the TheraBite^®^ interdental tool [34]. Treatment toxicities, including presence and extent of mucositis, oral cavity mucosa changes, and abnormalities were rated using (a) the Oral Cavity Assessment (OCA) [35] where a score of 7–10 is mild dysfunction and 15–18 is severe dysfunction and, (b) the NCI Common Terminology Criteria for Adverse Events (CTCAE) [36]. A grade of 0 indicates no symptoms and a grade of 4 indicates life-threatening consequences. Weight was tracked in pounds and pain level was recorded using responses to pain related question on the QLQ-C30 (no pain vs. pain, score 1 vs. 2–4). The European Organization for Research and Treatment of Cancer (EORTC QLQ-C30) and the Head and Neck Cancer Quality of Life module (EORTC QLQ-H&N35) were included as disease-specific, patient-reported quality of life measures.

At the baseline and 3-month time points only, all participants underwent a Modified Barium Swallow Study (MBS) with an Esophagram. MBS assessments were not conducted at later time points as the primary time point of interest was function at 3 months. Costs, additional radiation exposure, and patient burden from additional assessments led to the decision to limit MBS assessments to only the primary outcome measure time point. The MBS/Esophagram was conducted by experienced speech-language pathologists in conjunction with a radiologist. Participants were trialed on measured liquids sips, unmeasured liquid cup drinking, pudding, and cracker using Varibar^®^ products (Bracco Diagnostics Inc., Monroe Township, NJ) as well as trial of a barium tablet (E-Z-Disk™, Bracco Diagnostics Inc. Monroe Township, NJ). Participants were studied in lateral, anterior-posterior and right-anterior-oblique positions. A number of measures were calculated from the MBS/Esophagram. Dysphagia severity was rated using the 7-point Dysphagia Outcomes Severity Scale (DOSS) where 7 = normal diet, 1 = non-oral only [37]. The penetration–aspiration scale was calculated on all swallows with a score of 1 = normal, 2–5 = penetration, 6–8 = aspiration as assessed from the most impaired swallow across the study excluding the initial swallow [38]. Swallow physiology was assessed using a binary scale (present/absent) across 7 oral, 12 pharyngeal, and 6 esophageal phase deficits. Timing and efficiency of the oral and pharyngeal phases of swallow were evaluated using the Oral Pharyngeal Swallow Efficiency (OPSE) measures as calculated by dividing the percent of oropharyngeal residue by the duration of combined oral and pharyngeal phase transit time with a normal OPSE efficiency score of 50 or above [39]. MBS analyses were conducted by a number of experienced speech-language pathologists blinded to the study group. All raters underwent inter-rater reliability training beforehand by co-rating MBS studies with other raters until agreement was demonstrated.

#### Adherence to Treatment – Study Journal

For the exercise group only, data on therapy adherence was collected using a study journal which was completed daily during and, at least, up to 3 months post-CRT. Participants were asked to bring the study journal to each appointment. Adherence to treatment protocol was monitored by participants recording the number of days per week exercises was completed at least once, and the total number of exercise sets performed using an exercise checklist journal.

#### Exercise Group Protocol

Participants in the exercise group were instructed on a set of active exercises, completed twice daily, 7 days per week during CRT (exception CRT break week in week 4) and up to 3 months post-CRT. The set of exercises included oromotor strength/stretch exercises and swallow maneuvers. Also, each day participants completed the TheraBite^®^ exercise according to the 7-7-7 protocol (7 passive range of motion stretches, performed 7 times, repeated 7 times each day) [34]. See Table 1 for details on the swallow exercise protocol.

**Table 1:** Swallowing exercises performed by exercise group only* from start of treatment to 3 months post

Target	Exercises
Set 1: mandibular & neck range of motion exercises	Therabite: 7-7-7 protocol *both groups were instructed on the therabite
	Mouth open wide stretch. Repeat × 10
	Neck Stretch: sit on the palm of your right hand, bring left hand over your head and place just above your ear Stretch your head gently toward the left shoulder—hold 5 s—then drop chin down 5 times or until your chin gets down to your chest Move slowly. Then begin in middle position and do (1) 5 s hold with head extending back. Repeat to right by sitting on left hand
Set 2: labial range of motion exercises	Lip protrusion/retraction. Pucker and smile x 10
Set 3: lingual range of motion and strengthening exercises	Elevation, depression, lateralization, protrusion, anterior-posterior motion x 10 in each direction
	Retract tongue, hold 3 s, repeat × 10
Set 4: pharyngeal strengthening exercises	Masako Maneuver, repeat × 5
	Mendelsohn Maneuver, repeat × 5
	Effortful Swallow with mist bottle or liquids, repeat × 10
Swallowing	Swallow frequently throughout the day Continue eating and drinking by mouth, even when tube use starts Use spray mist bottle and other dry mouth products Stay hydrated

Frequency of practice: twice daily

Throughout the treatment period, CRT patients attended a weekly swallow therapy session which included continued instruction on the exercise protocol and swallow interventions as indicated. That is, instruction on compensatory swallow strategies and diet modifications were implemented, as indicated. A strong focus is that participants were encouraged to continue to eat and drink by mouth along with PEG use. For the remaining days of each week, participants completed the exercises independently and recorded compliance. The exercise protocol took approximately 20–30 min to complete twice daily.

#### Control Group Protocol

The control group received no direct SLP contact during treatment and did not complete any study exercise sets. However, all participants received a TheraBite^®^ prophylactically as per minimum standard of care set at the institution where the study was conducted. Following initial instruction on how to use the device, actual ongoing use of the device during and post-CRT was not monitored. No other prophylactic therapy was provided. If a participant in the control group was subsequently identified as having swallowing problems post-CRT by their medical care team (*n* = 4), they were provided with swallow therapy exercises as indicated. All were analyzed in the control group. Further information on these 4 patients is reported in results.

#### Sample Size

The sample size was determined by the statistician to be 30 participants in each group for a predicted power of 83% (2-tailed at *p* = .05) for a 1.0 scale point change on the FOIS between the two groups assuming a standard deviation of 1.3. This effect was selected as the smallest effect that would be important to detect, in the sense that any smaller effect would not be of clinical or substantive significance.

#### Statistical Methods

The analysis of this RCT was conducted in an intention to treat (ITT) approach. Clinicians entered all study findings on paper data entry forms which were scanned and saved. De-identified forms were then sent to a data entry company (Sosio Corporation, Inc., Glen Arm, Maryland) for independent double data entry verification. All statistical analyses were conducted by an independent statistician. The demographics and clinical factors at baseline were summarized overall and by each group and were compared with either Student’s t-tests or Fisher’s exact tests or Chi-square tests. The analysis of the primary variable (FOIS) and a number of the secondary variables (oromotor, incisal opening, weight, OCA, CTCAE, pain, DOSS, OPSE, FOSAD, Penetration and Aspiration scale) were computed with a generalized GEE models, by which the difference (odds ratio) of the two groups was estimated at each time points. The time to removal of PEG tube was defined from the end of CRT treatment to the time of PEG removal, censored at the date of death if the individual died with PEG on or at the date of last contact if the individual was lost to follow-up prior to the removal of the PEG tube. Kaplan–Meier estimates were used to plot the survival function and estimate the median time to PEG removal. A log-rank test was used to compare the time to removal between the two treatment groups. A *p* value of *p* ≤ 0.05 was deemed statistically significant. All analyses were done with SAS 9.4 and R 3.2.2.

## Results

### Participants

**Fig. 1:**
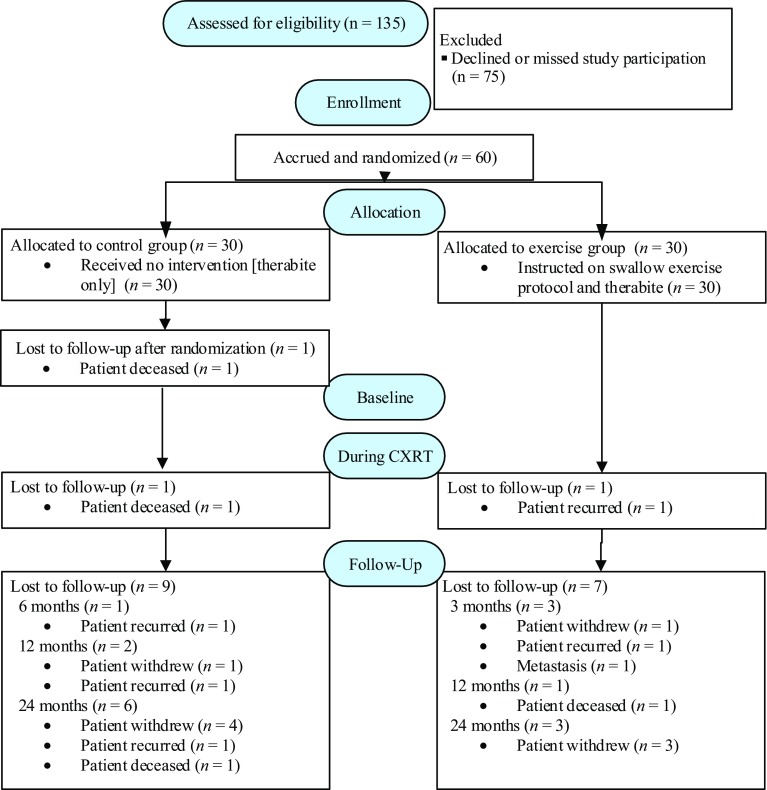
Consort Chart. Control: no intervention (exception: therabite). Exercise: combined swallow intervention study protocol and therabite

During the recruitment period, there were a total of 1,751 HNC patients admitted to the institution where the research was conducted. Following exclusion of patients with other HNC sites (*n* = 901), those scheduled to receive either surgical management or palliative care (*n* = 686), those who failed to meet the other study inclusion criteria (*n* = 29), and those who failed to consent or missed consenting (n = 75), 135 participants met eligibility criteria (Fig. 1). Of these 60 (45%) consented to participation. Demographics of the cohorts are detailed in Table 2. Comparison of the demographics between the groups revealed no significant differences. However, alcohol use appeared to be higher in the exercise group, with marginal statistical significance. Both groups consisted of predominantly male participants who presented with T2-3, N2 disease, primarily oropharyngeal tumors of the head and neck.

**Table 2:** Demographics

Characteristics	Overall	Control	N missing	Exercise	N missing	*P*-value
Age at enrollment						
Median(range)	56 (39, 79)	58(39, 79)	0	55(44, 78)	0	0.449
Gender: male, N(%)	54 (90%)	26(86.7%)	0	28(93.3%)	0	0.671
Weight at enrollment						
Median(range)	192(116, 330)	190(116, 315)	1	196(144, 330)	0	0.63
Alcohol use, N(%)	41(70.7%)	18(60%)	0	23(82.1%)	2	0.086
Tobacco use, N(%)	30(50.8%)	17(56.7%)	0	13(44.8%)	1	0.439
Stricture or web, N(%)	16(26.7%)	6(20%)	0	10(33.3%)	0	0.382
Tumor location, N(%)			0		0	
Larynx	6(10%)	2(6.7%)		4(13.3%)		0.671
Supraglottis	4	1		3		
Glottic	2	1		1		
Pharynx	54(90%)	28(93.3%)		26(86.7%)		
Oropharynx	49	25		24		
Hypopharynx	4	2		2		
Both	1	1		0		
T stage			0		0	0.883
1	9(15%)	5(16.7%)		4(13.3%)		
2	24(40%)	13(43.3%)		11(36.7%)		
3	22(36.7%)	10(33.3%)		12(40%)		
4	5(8.3%)	2(6.7%)		3(10%)		
Nodes, N(%)	50(83%)	28(93%)	0	22(74%)	0	0.79
N1		7(23%)		3(10%)		
N2a,N2b,N2c		21(70%)		17(57%)		
N3, N3b		0		2(7%)		

The majority of participants underwent a unilateral selective or modified radical neck dissection following the 3 months post-treatment PET/CT (controls, *n* = 18, 60%; exercise, *n* = 22,73%). Fewer participants had no neck dissection *n* = 8(27%); *n* = 5(17%) or bilateral modified radical neck dissections *n* = 2(7%), *n* = 2(7%), for controls vs. exercise groups respectively.

### Attrition and Protocol Compliance

Attrition occurred in both groups, and reasons for attrition at each time point are outlined and shown in Fig. 1. Post-CRT, 28 control, and 29 exercise participants were available for reassessment. At each subsequent time point, further attrition occurred, although 83% were still available for analysis at 12 months in both groups and over 68% with data at 24 months. All participants in the control group were seen for assessments only, as per protocol, except for 4 participants who were referred for swallowing management after the 3 month MBS study which revealed dysphagia requiring intervention. These 4 participants received between 4 and 6 therapy sessions after the 3-month assessment time point. Therapy provided was not the same as the exercise protocol in this current study but rather exercises were targeted based on the participants’ oropharyngeal phase disorder(s). The amount of treatment provided was limited. Out of these 4 participants, two had 4 swallow therapy sessions, 1 had 5 sessions, and 1 had 6 sessions. All 4 of these control participants were study completers and were followed up to 24 months post-CRT.

### Primary Outcome Measure

The greatest difference (10% difference) in the proportion of patients with dysphagia (FOIS 1-5) was observed between groups at 3 months post-CRT (Table 3); however, this was not statistically significant (odds ratio [OR], 1.5; 95% confidence interval, CI, 0.5–4.8; *p* = 0.49) (Table 4). There was no significant difference at any other time points.

**Table 3:** Summary data^a^ of Clinician and Patient-Reported Outcomes across time points

Parameter	Baseline/pre-treatment		3 months post CRT		6 months post CRT		12 months post CRT		24 months post CRT	
	Control	Exercise	Control	Exercise	Control	Exercise	Control	Exercise	Control	Exercise
*Oral/Non*-*oral Intake*										
FOIS 1-5^b^	10%	0%	60%	50%	33%	27%	4%	12%	0%	0%
* PEG insitu* ^c^	100%	100%	61%	76%	18%	29%	4%	14%	0%	3%
* Food Category 2*-*5* ^*d*^	28%	13%	60%	68%	42%	39%	15%	24%	11%	14%
* Fluid Category2*-*5* ^*e*^	0%	0%	12%	9%	4%	9%	0%	4%	0%	0%
Oromotor and toxicities										
* Oromotor assessment* ^*f*^	16%	13%	24%	9%	33%	5%	12%	8%	1%	9%
* Incisal opening*	46.4(9.5)	50.7(5.9)	41.9(8.4)	43.2(8.5)	39.9(7.3)	43.9(8.5)	43.8(7.0)	46.7(7.4)	44.1(6.3)	48.6(8.8)
* OCA* ^*g*^	32%	43%	5%	7%	4%	5%	8%	4%	11%	14%
CTCAE/CE^h^	0%	4%	68%	93%	20%	42%	12%	16%	0%	10%
CTCAE/FS^i^	0%	7%	58%	86%	28%	37%	8%	4%	0%	10%
Pain^j^	70%	76%	53%	46%	58%	43%	39%	33%	33%	33%
Weight (pounds)	195(42)	203(44)	170(35)	174(20)	173(31)	167(24)	181(33)	177(25)	191(33)	180(27)
QOL: EORTC^k^										
*QLQ*-*C30*										
*Function*	77.9(18.1)	76.3(16.5)	79.7(18.6)	85.2(12.8)	88.5(13.4)	82.1(22.7)	89.2(10.7)	89.3(15.7)	95.3(5.4)	92.3(9.9)
*Global health*	73.9(17.6)	71.0(15.0)	74.0(17.4)	84.0(10.9)	81.6(15.9)	77.8(22.4)	85.7(13.5)	80.3(17.4)	89.2(13.7)	88.4(12.8)
*Symptom*	18.8(14.5)	21.0(12.1)	20.4(11.9)	14.0(14.2)	13.3(10.5)	13.4(13.0)	10.7(9.7)	10.1(12.5)	7.1(6.7)	8.5(7.6)
* QLQ*-*H&N35*	23.1(16.7)	15.1(9.0)	27.1(10.5)	23.2(14.0)	22.6(12.0)	17.8(11.3)	16.2(8.5)	17.5(16.0)	10.8(7.6)	14.4(10.7)
* HN Swallowing*	23.7(33.7)	11.1(13.5)	20.4(17.8)	16.0(22.3)	15.3(17.1)	18.9(23.6)	14.9(13.0)	12.3(22.6)	8.8(7.5)	11.1(12.1)
* HN Social eating*	17.9(27.0)	6.7(10.1)	27.9(23.2)	11.4(11.3)	13.5(15.9)	23.0(30.2)	13.6(11.1)	18.3(25.9)	3.6(6.8)	12.0(18.8)

^a^ It shows percentage for binary outcomes and mean(SD) for continuous outcomes

^b^ Functional Oral Intake Scale (FOIS) expressed as proportion of group receiving a rating of 1-5 indicating more impaired and more restricted diet level

^c^ Proportion of group with a Percutaneous Gastrostomy (PEG) in situ

^d^ Proportion of patients managing food consistencies other than normal

^e^ Proportion of patients managing fluid consistencies other than normal

^f^ Percentage of patients with total score ≤ 65 indicating impaired function

^g^ Oral Cavity Assessment (OCA). Percentage of patients reporting oral symptoms

^h^ Common Terminology Criteria for Adverse Events (CTCAE) Clinical exam (C/E). Percentage demonstrating mucositis (scores 1–3)

^i^ Common Terminology Criteria for Adverse Events (CTCAE) Function/symptom (F/S). Percentage presenting with oral mucosa erythema, ulcerations (scores 1-3)

^j^ Percentage reporting pain within last week as determined from QLQ-C30 Q9 (score 1 no pain vs. 2–4 pain)

^k^ Eastern Organization for Research and Treatment of Cancer (EORTC: QLQ-C30, QLQ-H&N35) quality of life scores expressed as mean and standard deviation

**Table 4:** Analysis^1^ of Clinician and Patient-Reported Outcomes across time points

Parameter	Baseline/pre-treatment		3 months post CRT		6 months post CRT		12 months post CRT		24 months post CRT	
	OR/Difference	*P*-value	OR/Difference	*P*-value	OR/Difference	*P*-value	OR/Difference	*P*-value	OR/Difference	*P*- value
Oral**/**Non-oral Intake										
FOIS 1-5^2^	–	1.0	1.50(0.47, 4.77)	0.49	1.33(0.38, 4.72)	0.66	0.29(0.03, 3.03)	0.30	–	1.0
*PEG insitu* ^*3*^	–	1.0	–	0.68	–	0.55	–	0.36	–	1.0
*Food Category 2*-*5*	2.5(0.7, 9.4)	0.18	0.7(0.2, 2.3)	0.56	1.1(0.4, 3.6)	0.86	0.6(0.1, 2.4)	0.44	0.8(0.1, 5.3)	0.81
*Fluid Category 2*-*5*	–	1.0	–	0.61	–	0.61	–	0.49	–	1.0
Oromotor and toxicities										
*Oromotor assessment*	1.2(0.3, 5.6)	0.78	3.2(0.6, 17.6)	0.19	10.0(1.1, 88.5)	**0.04**	1.5(0.2, 9.87)	0.67	1.2(0.2, 9.3)	0.88
*Incisal opening*	3.7(−0.6, 8.0)	0.09	1.2(−3.3, 5.7)	0.60	3.7(−0.7, 8.1)	0.10	2.2(−1.7, 6.2)	0.26	4.6(0.3, 8.9)	**0.04**
*OCA*	1.6(0.5, 4.9)	0.42	1.4(0.1, 24.2)	0.82	1.3(0.1, 22.8)	0.84	0.5(0.04, 5.7)	0.56	1.4(0.2, 9.6)	0.72
CTCAE/CE^4^	–	1.0	6.0(0.6, 57.1)	0.12	2.9(0.8, 11.1)	0.12	1.4(0.3, 7.0)	0.69	–	0.49
CTCAE/FS^5^	–	0.49	4.1(0.7, 22.8)	0.11	1.4(0.4, 4.9)	0.62	0.6(0.05, 6.1)	0.63	–	0.49
Pain	0.7(0.2, 2.6)	0.62	1.3(0.3, 5.6)	0.71	1.9(0.6, 6.1)	0.30	1.3(0.4, 4.4)	0.69	1.0(0.3, 4.0)	1.0
Weight (pounds)	7.7(−13.9, 29.3)	0.49	2.2(−13.9, 18.2)	0.79	3.8(−12.3, 19.9)	0.64	−1.5(−17.8,14.8)	0.86	−4.8(−20.9, 11.3)	0.56
QOL: EORTC										
*QLQ*-*C30*										
*Function*	−2.8(−12.2, 6.6)	0.56	5.2(−5.3, 15.7)	0.33	−4.7(−15.2, 5.8)	0.38	0.6(−7.2, 8.3)	0.89	−0.9(−6.7, 4.8)	0.75
*Global health*	−2.3(−11.3, 6.7)	0.62	10.1(−0.1, 20.3)	**0.05**	−3.4(−14.5, 7.8)	0.56	−5.2(−14.1, 3.7)	0.25	−0.1(−8.7, 8.5)	0.98
*Symptom*	3.2(−4.0, 10.4)	0.38	−6.0(−14.5, 2.5)	0.17	−0.5(−7.1, 6.0)	0.87	−0.6(−7.0, 5.7)	0.84	0.11(−4.8, 5.0)	0.97
*QLQ*-*H&N35*	−6.2(−13.7, 1.3)	0.11	−1.8(−9.7, 6.0)	0.65	−4.6(−11.0, 1.8)	0.16	0.2(−6.9, 7.3)	0.95	2.8(−2.9, 8.5)	0.33
*HN Swallowing*	−11.5(−28.1, 5.1)	0.17	−1.4(−15.9, 13.2)	0.85	3.6 (−8.2, 15.0)	0.57	−2.8(−13.3, 7.7)	0.60	1.5(−5.0, 8.0)	0.65
*HN Social eating*	−9.6(−22.4, 3.3)	0.14	−12.8(−25.8, 0.2)	**0.05**	8.8 (−4.9, 22.5)	0.21	3.8(−7.5, 15.1)	0.51	7.0(−2.0, 16.0)	0.13

1. Bold font indicates significance at *p* ≤ *0.05.* The outcomes are the same as described in Table 2. For binary outcome, odds ratio and 95% CI are presented. For continuous outcomes, differences and 95% confidence intervals were presented

2,3,4,5. For some variables at certain time points where odds ratio could not be estimated or applicable, fisher’s exact tests were used

### Secondary Outcome Measures

The nature of foods or liquids managed were not found to be significantly different at any time point (Table 4). The proportion of patients requiring a modified diet in the control and exercise groups was low at baseline (28, 13%) with most patients tolerating a normal diet. Patients’ diet level declined during and up to 3 months post-treatment with a high proportion of patients in both groups restricted to a modified diet (60, 68%). Diet level improved somewhat at 6 months post-treatment (42, 39%) with return to baseline by 24 months post-treatment (11, 14%). Very few participants in either group required modified liquids (Table 3).

**Fig. 2:**
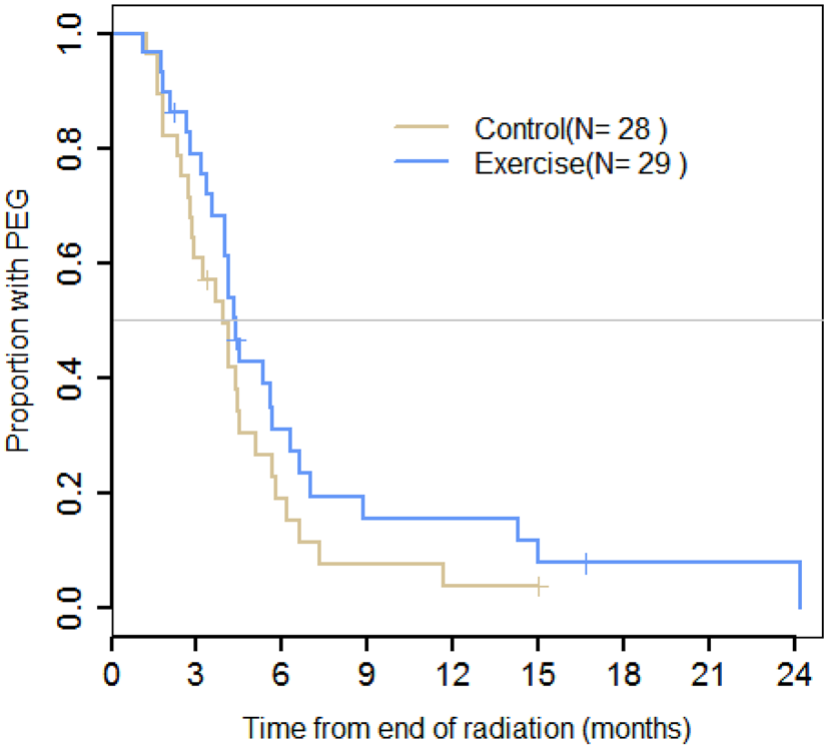
Status of PEG tube PEG placement duration

The Kaplan–Meier estimates of survival function revealed no significant difference (*p* = 0.17) in the time to removal of PEG tubes, with median duration to removal being 3.9 months (95% CI, 2.8–5.7) for the control group, and 4.4 months (95% CI, 4.0–6.6) for the exercise group (Fig. 2). It was noted in the exercise group that one participant developed stricture and required 16 dilatations. That participant retained the PEG until just prior to his 24-month time point. There was no significant difference detected between the groups in terms of proportions of patients with PEG tubes in place at any time point (Tables 3 and 4). Similarly, there were no differences observed between groups for assessments of mucositis, erythema, pain, or weight (Tables 3 and 4).

Some isolated differences were observed between groups on some parameters, with oromotor function found to be better in the exercise group at 6 months (OR 10.0, 95% CI, 1.1–88.5; *p* = 0.04) and greater incisal opening in the exercise group at 24 months (difference in score, 4.4, 95% CI, 0.3–8.9; *p* = 0.04) (Table 4). Outcomes from the EORTC QLQ-C30 and QOL-H&N35 revealed no significant differences between groups except for global health (difference in score, 10.1, 95% CI, −0.1–20.3; *p* = 0.05) and HN social eating (difference in score, −12.8, 95% CI, −25.8–0.2; *p* = 0.05) at 3 months (Table 4).

Results from the physiological swallow assessments at baseline and 3 months found OPSE overall swallow efficiency and percent of bolus swallow findings to be statistically significantly better in the exercise group at 3 months post-treatment. The odds ratio of having better swallow efficiency (OPSE total score > 50) was 4.2 (95% CI, 1.1–15.7; *p* = 0.04) at 3 months and the odds ratio of having normal OPSE % bolus (>95%) was 4.0 (95% CI, 1.0–5.5; *p* = 0.05) at 3 months post-treatment (Table 5). There were significantly fewer pharyngeal phase impairments in the exercise group at 3 months and a trend for shorter pharyngeal transit times (Table 5). The odds ratio of having normal pharyngeal score is 6.9 (95% CI, 1.7–28.1; *p* = 0.007) for the exercise group compared to the control group.

**Table 5:** Analysis^a^ of swallow physiology at baseline and 3 months

Parameter *MBS*	Baseline**/**pre**-**treatment			3 months post CRT		
	Control (%)	Exercise (%)	*P*-value	Control (%)	Exercise (%)	*P*-value
Dysphagia Outcome Severity Scale ^b^	11	7	0.61	25	40	0.29
OPSE OTT (seconds)^c^	18	11	0.45	21	15	0.62
OPSE PTT (seconds)^d^	29	14	0.20	46	20	0.08
OPSE bolus swallowed^e^	43	25	0.16	50	20	**0.05**
OPSE total score^f^	29	11	0.10	46	15	**0.04**
Oral phase impairments^g^	25	10	0.16	29	10	0.13
Pharyngeal phase impairments^g^	61	41	0.15	83	42	**0.007**
Esophageal phase impairments^g^	56	38	0.19	50	47	0.87
PAS Penetration 2-5 ^h^	29	10	0.09	38	21	0.33
PAS Aspiration 6-8^i^	7	7	1.0	9	0	0.49

^a^ Fisher’s exact test was used at each time points. Other variables used GEE models to get p-values. Bold font indicates significance at *p* ≤ *0.05*

^b^Dysphagia Outcome Severity Scale (DOSS), dichotomized as 1–5 vs. 6–7, shows percentage of abnormal (1–5)

^c^OPSE: OTT Oral Transit Time: shows percentage of abnormal (>1.0 s)

^d^OPSE: PTT Pharyngeal Transit Time: normal, percentage of abnormal (>1.0 s)

^e^OPSE% bolus swallowed: ≤95% with trace or minimal residue

^f^OPSE total score: percentage of abnormal (≤50 total OPSE)

^g^Oral, pharyngeal and esophageal phase impairments. Percentage of patients with at least one problem in this phase

^h^Penetration–Aspiration Scale (PAS) Penetration: Percentage of patients with penetration score of 2–5

^i^Penetration–Aspiration Scale (PAS) Aspiration: Percentage of patients with aspiration score of 6–8

### Adherence Data

**Fig. 3:**
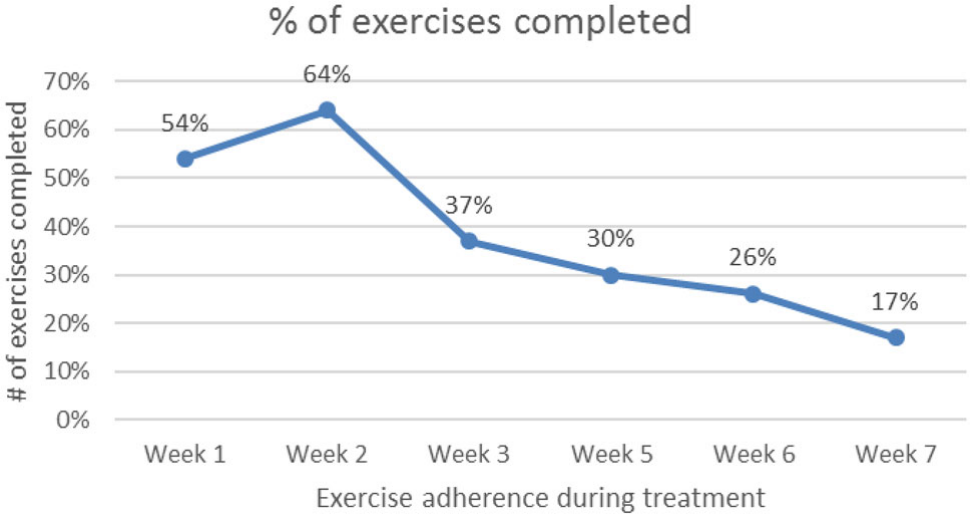
Adherence

**Fig. 4:**
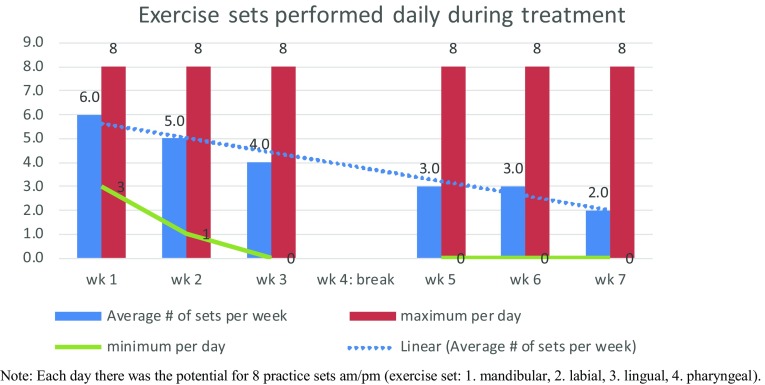
Exercise sets practiced daily during treatment. Each day there was the potential for 8 practice sets am/pm (exercise set: 1. mandibular, 2. labial, 3. lingual, 4. pharyngeal)

Study journals were fully complete for 66% (*n* = 19) of the exercise group. The remainder had completed journal entries sporadically. On average, participants in the exercise group completed some therapy 4 days per week. During weeks 1 and 2, the percentage of exercises completed was moderate with 54 and 64%, respectively. At week 2, adherence was fairly good with 56% practicing every day and 100% practicing at least 4 times per week. At week 5, adherence was moderate with 41% practicing every day and 53% practicing at least 4 times per week. For weeks 3, 5, 6, and 7 the percentage of exercise was low at 37, 30, 26 and 17%. Across the 6 weeks, 28% completed 40% or more of their daily exercises with 72% completing less than 40% (see Fig. 3). Further detailed analysis of adherence confirms a gradual decline of daily exercise practice over the 7 weeks of treatment. Participants performed an average of 6 exercises daily during treatment week 1 declining to an average of 2 per day by week 7 (see Fig. 4).

## Discussion

Although it is recognized that the sequelae of undergoing CRT and experiencing the acute toxicities associated with treatment negatively impact the ability to participate in active therapy, participants in the current study demonstrated fairly good adherence to the treatment protocol. However, as expected, adherence dropped to its lowest point by week 5 of CRT in the current study. It is commonly reported that acute toxicities reach their peak impact at 5 weeks (mean number of weeks) which is consistent with several recent studies’ findings of partial or moderate exercise protocol adherence [26, 31, 40]. Fairly good adherence to prophylactic therapy protocols in the early stages during CRT, with greater attrition later in treatment, has also been reported in other recent studies [22, 27, 31]. However, despite a fairly good result for level of adherence to the exercise protocol, there was limited evidence to support that the exercise group achieved superior outcomes. With respect to the primary outcome variable of functional diet level, differences between the groups only approached significance at 3 months post-treatment.

At all time points, including the primary time point at 3 months, there was no difference between the groups regarding FOIS scores, with both groups demonstrating 100% of individuals returning to a largely non-modified diet (FOIS 6–7) by 24 months. Carnaby-Mann et al. [22] and Mortensen et al. [31] also reported a lack of difference in functional diet level. In an RCT, van den Berg et al. [40] found no differences in diet levels, although this may have been because the swallow exercise protocol was primarily instruction on compensatory swallow strategies rather than physiologically based swallow exercises. In prior research, Kotz et al. [26] is the only study to date to find significantly better FOIS results at 3 and 6 months. It is unclear exactly why that study found different results. One factor may be that the Kotz et al. [26] study included the super-supraglottic swallow in their treatment protocol, unlike the current study or others. It is possible that targeting airway protection and swallow safety may have contributed to better diet outcomes in their study.

The current study also found no differences in duration of PEG tube dependency. In both groups, most participants had their PEG removed between 3 and 6 months post completion of treatment. Only 18 and 29% of patients in the control and exercise group required a PEG at 6 months post-treatment. Therefore, PEG dependence was not a dominant issue in this study. Kotz et al. [24] reported a similar median time to PEG removal of 3 months post-treatment in their cohort and also failed to find any between group differences [26]. Mortensen et al. [31] also found no differences between their groups for PEG duration. In contrast, the study by van der Molen et al. [28] found significantly lower PEG tube use in their exercise groups compared to a historical study cohort (used as control); however, the lack of a randomized control study design limits direct comparison to the current data.

Although there was no consistent positive effect observed across time, oromotor function was statistically significantly better at 6 months in the current exercise group. This may relate to the active oromotor exercises completed as part of daily therapy within the treatment protocol. Similar oral exercises were performed in the treatment arm of the Carnaby-Mann et al. [22] study and better preservation of muscle size and composition (i.e., genioglossus, hyoglossus, mylohyoid) were found at 6 months post-CRT.

Incisal opening was also greater in the exercise group at 24 months. Overall, both groups had well-preserved incisal opening without trismus throughout the study. This finding may be because both groups were provided with a TheraBite^®^ as per this settings’ standard of care. Additionally, the significantly greater incisal opening in the exercise group at 24 months may be attributed to the consistent and encouraged use of the TheraBite^®^ versus the self-directed practice in the control group. Loss over time in the exercise group was only 2 mm, which was comparable to findings observed by Carnaby-Mann et al. [22] who observed a 1.6 reduction in their treatment arm.

Although there was no statistically significant difference in levels of functional oral intake, a significant association was found between the active oral exercise and pharyngeal phase physiology at 3 months post-treatment, with significantly less pharyngeal phase impairments and overall better swallow efficiency observed in the exercise group. This result was similar to the findings of Carnaby-Mann et al. [22]. Possible reasons for positive results in this current study may be due to the targeting of pharyngeal phase structural movements in the exercise protocol including the Masako (tongue hold), Mendelsohn and the effortful swallow. Kotz et al. [26] also used the effortful swallow, Masako, and Mendelsohn and although they reported better FOIS scores in the exercise arm at 3 months post-treatment no data on swallow physiology was provided. In contrast, in the Carnaby-Mann study [22], the high-intensity exercise group performed the falsetto, tongue press, hard swallow and TheraBite^®^ (jaw resistance) exercises, whereas Mortensen et al. [31] utilized the tongue hold, gargle, tongue range of motion, jaw opening and circular movements, laryngeal range of motion, Shaker and falsetto exercises. Exercise protocol differences may have contributed to inconsistent results across existing studies.

Considering the relative lack of functional differences and similar experiences regarding acute toxicities and weight loss, it was not unexpected that participant assessments of level of function on the QOL-C30 and QOL-H&N35 failed to detect statistically significant differences between the two groups on most subscales. Similar to Mortensen et al. [31], the global health score on the QOL-C30 at 3 months in the current study showed a statistically significant difference in favor of the exercise group. However, this was not maintained at 6, 12, or 24 months. Likewise, in the current study, QOL-H&N35 social eating scores were borderline statistically significant in the exercise group at 3 months post-CRT but not at any other time points. Similarly, recent studies using the MD Anderson Dysphagia Inventory (MDADI) found better swallowing-related QOL scores in patients performing prophylactic swallowing exercises [4, 41, 42].

Strengths of the current study include its randomized controlled study design, inclusion of both physiological and functional swallowing parameters as well as patient and clinician-reported outcome measures, and its substantial follow-up period (to 24 months). Adherence to the study protocol was good, and attrition was low. However, a major issue for all research into the benefits of prophylactic swallow protocols is that there is little agreement on the nature and type of therapy program participants should follow. As discussed, all studies to date have designed and developed treatment protocols with different exercises completed at different levels of intensity and for varying durations during and after CRT. The current study protocol was developed in 2004 when there was little other published evidence. It was designed to target oromotor function and maintaining function of components of the swallow. However, it did not specifically target airway protection exercises. It should also be noted that participants were only encouraged to complete exercises up to 3 months. Whether or not continuing to exercise in the later month’s post-treatment would have benefits for long-term outcomes cannot be determined from this data. Another potential limitation may be the use of PEG tubes. PEG tube use was only initiated once the patient was deemed to be unable to support their nutritional needs orally. However, given the current controversy over whether or not the presence of a PEG during treatment negatively affects swallowing outcomes this may or may not have had an effect on study outcomes. The lack of physiological data at time points beyond 3 months is also acknowledged to limit our understanding of the long-term benefits of treatment. However, as long-term outcomes were only a secondary aim, and considering costs, additional radiation exposure, and patient financial burden, these assessments were not conducted. Future studies may consider performing Flexible Endoscopic Evaluation of Swallow (FEES) as a complimentary method of evaluating the pharyngeal swallow [43] when conducting assessments at multiple interval time points post-treatment. Studies have identified the FEES to have high sensitivity and specificity compared to MBS and can be used as a complementary diagnostic tool in the identification of pharyngeal dysphagia without the increased risk of additional radiation exposure [44, 45].

The strict inclusion criteria set for this study, and the fact that only 45% of eligible participants consented to participation, also means that the current cohort cannot be considered representative of all patients who may benefit from prophylactic swallow therapy. Finally, there was a degree of partial or missing data points across study time points, as is a factor of any long-term study. While the GEE model assumes missing completely at random (MCAR), it may not be as robust when the data were not MCAR. Further, the confidence intervals of some outcomes are relatively large, reflecting effects from either imprecision of the measurement or too small sample size.

## Conclusions

Despite some positive early physiological changes, functional swallowing outcomes at 3 months and up to 24 months did not demonstrate significant benefits. Differences in exercise protocols across studies and their impact on patient outcomes cannot be currently discounted. These issues exemplify the need for further research studies designed to examine appropriate prophylactic swallowing exercises implemented with adequate frequency, intensity, and long-term practice for maximum functional gain and recovery.
